# Genomic insights into diversity, antimicrobial resistance and virulence of *Glaesserella parasuis* from diseased swine in Peru

**DOI:** 10.3389/fmicb.2025.1678153

**Published:** 2025-09-23

**Authors:** Luis Alvarez-Vega, Karolina S. M. De La Cruz-Monroy, Arturo Garcia-Barraza, Dennis Carhuaricra, Jose L. Llaja-Bernedo, Liseth L. Huamancha-Pulido, Alejandra Medina-Gonzales, Andre F. Sedano-Sanchez

**Affiliations:** Laboratorio Veterinario Lasser-Jallavet, Lima, Peru

**Keywords:** *Glaesserella parasuis*, whole-genome sequencing, phylogeny, antibiotic resistance genes, Oxford Nanopore sequencing

## Abstract

*Glaesserella parasuis* is an important bacterial pathogen and the etiological agent of Glässer’s disease, which causes substantial economic losses on swine farms worldwide. Although large scale genome studies have been carried out in the North hemisphere, few genomes from South America are available limiting our knowledge of the full genetic diversity of this bacteria. In this study, seventeen clinical *G. parasuis* isolates from diseased swine in Peru where whole genome sequenced and compared with publicly available genomes. We identified seven distinct sequence types and five serotypes being more prevalent: the emergent ST454 and novel ST726 and serotypes 7 and 12. Although most isolates did not carried any antimicrobial resistance gene (ARG) we identified a large cassette of ∼50 kb carrying nine different ARGs (*aac(3)-IV, aph(3′)-Ia, aph(3′)-Ib, sul2, bla*_*ROB–*1_ *tetB, lnuH, estT* and *floR*) including six tandem repeats of *estT* and *floR* genes. We also identified two coexisting small plasmids harboring *tetH, estT* and *bla*_*ROB–*1_ in two isolates. Most of the isolates (88.2%, 15/17) harbored at least one group 1 *vtaA* virulence gene. The *G. parasuis* diversity was segregated in two clades (C1 and C2) by phylogenetic and gene content analysis. We identified several genes overrepresented in both clades, however three metabolic operons (*glnHMPQ, hisGDCBHAFI, thiDEM*) were almost exclusive of C2 strains suggesting that C1 strains are auxotrophs for histidine and thiamine important nutrient for bacterial survival which may impact in the adaptation of these strains to the host niche. Our results suggest a high diversity of *G. parasuis* strains circulating in Peru, the presence of mobile elements carrying multiple ARGs underscores the need for continuous surveillance of this pathogen within the Peruvian swine industry to identify potential vaccine candidates and formulate better control programs.

## 1 Introduction

*Glaesserella parasuis*, an early colonizer of the upper respiratory tract of piglets, is an important bacterial pathogen affecting pig farms (Costa-Hurtado et al., 2019). *G. parasuis* causes Glässer’s disease in weaned pigs, producing polyserositis, arthritis and meningitis (Aragon et al., 2019). This disease can result in high mortality and economic losses for pig production (Zhang et al., 2014).

Control of Glässer’s disease depends on accurate typing of pathogenic strains. Therefore, serotyping has usually been used as a typing method, with fifteen serotypes based on capsular polysaccharides (Kielstein and Rapp-Gabrielson, 1992; Tadjine et al., 2004). The serotypes of *G. parasuis* have been classified into three groups according to their virulence. Serotypes 1, 5, 10, 12, 13 and 14 are classified as highly virulent; serotypes 2, 4, 8 and 15 as moderately virulent; and serotypes 3, 6, 7, 9 and 11 as avirulent (Kielstein and Rapp-Gabrielson, 1992). However, this classification was determined using only reference strains of each serotype, so the relationship between serotype and virulence in *G. parasuis* is not well established in field isolates (Aragon et al., 2010). As an alternative, Multi-Locus Sequence Typing (MLST) has been employed, offering better strain discrimination compared to serotyping (Mullins et al., 2013) revealing a high genetic diversity of *G. parasuis* with almost 900 sequence type (ST) reported (PubMLST database, accessed in April 2025). In recent years, whole genome sequencing (WGS) has been used for pathogen epidemiology surveillance because it offers greater discrimination between isolates by allowing the determination of relevant information related to phylogeny, ST, putative virulence factors and antimicrobial resistance genes (ARGs) (Wan et al., 2020). The detection of several virulence-associated genes has been proposed to determine the virulence of *G. parasuis* isolates (Oliveira and Pijoan, 2004; Pina et al., 2009; Sack and Baltes, 2009; Martínez-Moliner et al., 2012). The virulence-associated trimeric autotransporter (*vtaA*) genes have been reported as the most relevant virulence factors in *G. parasuis* (Pina et al., 2009), mainly group 1 *vtaA* genes (Costa-Hurtado et al., 2012). A high number of different ARGs have been reported by WGS studies including *tetB, sul2, aph(3’)-Ia, aph (6)-Id* and *bla*_*ROB–*1_ which can be transferred by mobile genetic elements (Wan et al., 2020; Gong et al., 2024).

Numerous genotypes and serotypes of *G. parasuis* have been identified, yet no clear correlation has been established between virulence and its phenotypic or genotypic characteristics (Gong et al., 2024). Although autogenous vaccines have successfully reduced mortality, vaccine failures remain common due to limited cross-protection (Macedo et al., 2015). Therefore, comprehensive studies on the genetic diversity of *G. parasuis* are essential for the development of more effective vaccines. Despite the significant expansion of the swine industry in South America, research addressing the genetic diversity of *G. parasuis* in the region remains limited (Espíndola et al., 2019). This study aimed to gain insights into the diversity of *G. parasuis* circulating in Peru, identifying the STs, serotypes, ARGs and virulence factors using WGS to provide information for control of the disease in the swine production.

## 2 Materials and methods

### 2.1 Isolation and identification

Seventeen *G. parasuis* isolates analyzed in this study were obtained from clinical samples of pigs diagnosed with Glässer’s disease, submitted to the Laser Veterinary Laboratory (Lima, Peru) between 2021 and 2024. The samples were collected from nine farms (coded as A to I) located on the central coast of Perú. Due to privacy restrictions, the specific farm locations and pig breeds are not disclosed. Detailed information on the specific isolation organs and the farm of origin for each sample is provided in Supplementary Table 1.

*G. parasuis* isolation was performed following Markey et al. (2013). Briefly, tissue sections were taken aseptically and cultured on tryptic soy agar (Neogen, USA) with 5% sheep blood and a streak of *Staphylococcus aureus* ATCC 25923 (Microbiologics, USA), which served as a source of nicotinamide adenine dinucleotide (NAD), a necessary growth factor. The samples were then incubated at 37 °C in a microaerophilic environment (5% CO2) for 18–24 h. Small, translucent, non-hemolytic colonies exhibiting satellite growth near the *S. aureus* streak were considered presumptive *G. parasuis* (Garrity et al., 2005). A loopful of presumptive *G. parasuis* colonies from each sample was suspended in 500 μL of sterile phosphate-buffered saline (PBS). Genomic DNA was extracted using the Real PCR RNA/DNA Magnetic Bead Kit (IDEXX Laboratories, USA) following the manufacturer’s instructions. Species confirmation was performed by qPCR using primers targeting the *infB* gene (forward primer: 5′-CGACTTACTTGAAGCCATTCTTCTT-3′, reverse primer: 5′-CCGCTTGCCATACCCTCTT-3′) (Turni et al., 2010). The reaction was performed using 1X GoTaq qPCR Master Mix (Promega Corporation, USA), 1 μM of each primer, 3 μl of template DNA and molecular biology-grade water (neoFroxx GmbH, Germany) to a final volume of 25 μL. Cycling conditions were as follows: initial denaturation at 95 °C for 2 min; 40 cycles at 95 °C for 15 s, 58 °C for 15 s and 72 °C for 50 s; and a final melting curve analysis at 65 °C–95 °C with increments of 0.5 °C every 2 s. PCR reactions were performed using the CFX96 Touch Real-Time PCR System (Bio-Rad Laboratories, USA).

### 2.2 Whole genome sequencing

The DNA quality (260/280 and 260/230 ratios) was assessed using a Nabi UV/Vis Nano Spectrophotometer (MicroDigital Co., Ltd, Korea) and quantified using a Quantus fluorometer (Promega, USA). DNA Library was prepared using the Native Barcoding Kit 24 V14 (SQK-NBD114.24, Oxford Nanopore, UK) following the ligation protocol. Briefly, DNA was repaired using FFPE DNA Repair Mix (M6630L, New England Biolabs, USA) and A-tails were added using the Ultra II End Repair/dA-Tailing Module (E7546L, New England Biolabs, USA). Native barcodes (SQK-NBD114.24, Oxford Nanopore, UK) were ligated to DNA fragments using the Blunt/TA Master Mix (M0367L, New England Biolabs, USA). The samples were then cleaned with magnetic beads and 80% ethanol to remove DNA repair enzymes and excess barcodes. A final cleanup of the DNA library was performed with beads and long fragment buffer (SQK-NBD114.24, Oxford Nanopore, UK), and DNA was eluted in 15 μL of elution buffer. Sequencing was performed using MinION R10.4.1 flow cells on the MinION Mk1C instrument and was run for approximately 18 h. After each use, flow cells were washed using the Flow Cell Wash Kit (EXP-WSH004, Oxford Nanopore, UK) and stored at 4 °C.

The sequenced reads were basecalled with Dorado v0.9.1 using the dna_r10.4.1_e8.2_400bps_sup@v4.3.0 model. The reads were trimmed using Porechop v0.2.4^1^ to remove adapters and Filtlong v0.2.1^2^ and to filter lengths greater than 2000 bp for downstream assembly. *De novo* assembly was performed using the Autocycler pipeline^3^, which employs a consensus strategy by integrating results from multiple long-read assemblers, including Flye (Kolmogorov et al., 2019), Canu (Koren et al., 2017), miniasm (Li, 2016), and NextDeNovo (Hu et al., 2024). This approach has been shown by its authors to reduce error rates and improve structural accuracy (Wick et al., 2025). The assemblies were polished by using Medaka 2.0.1 to further correct residual errors. QUAST 5.2.0 (Gurevich et al., 2013) was employed to check the assembly quality. Genomes were annotated using Prokka 1.14.5 (Seemann, 2014) with default parameters. Species assignment was further validated by Average Nucleotide Identity (ANI) analysis against the reference *G. parasuis* strain Nagasaki (Accession: NZ_CP018034) using the FastANI software (Jain et al., 2018). All genomes showed ANI values greater than 95% relative to the reference, confirming their classification as *G. parasuis*.

### 2.3 In silico genotyping of *G. parasuis*

The *in silico* Serotyping was performed using ABRICATE v.1.0.1^4^ against a custom database that included the genes proposed by Howell et al. (2015) and Jia et al. (2017) to detect all 15 serotypes. We applied thresholds of ≥90% minimum identity and ≥80% minimum coverage to the ABRicate output for sequence identification, following the criteria used by Gong et al. (2024). The ST of the isolates was determined using mlst v.2.23.0 tool^5^ with up-to-date *Glaesserella parasuis* scheme and profiles downloaded from the pubMLST database^6^. Fasta files of isolates with novel alleles or allelic profiles were submitted to the PubMLST database for assignment of a ST number. Novel ST were added to the pubMLST database along with their corresponding metadata.

### 2.4 Phylogenetic analysis

To elucidate the phylogenetic relationship of Peruvian isolates within a global context we included a dataset of 216 *G. parasuis* genomes from the study by Mugabi et al. (2023), available with the Bioproject number PRJNA749326. A total of 233 genomes were aligned against a reference *G. parasuis* strain Nagasaki (Accession: NZ_CP018034) using Parsnp v2.1.3 (Kille et al., 2024). The recombinant regions were removed, and a maximum likelihood tree was calculated with IQTREE-2 v2.4.0 (Minh et al., 2020) using the general time reversible-gamma model with 1000 ultrafast bootstraps. The phylogenetic tree was visualized using the ggtree v3.14 package (Yu, 2020) in R v4.4.2.

### 2.5 Analysis of the pangenome

Panaroo v1.5.1 (Tonkin-Hill et al., 2020) was used to generate the pangenome of all 234 *G. parasuis* genomes with the following flags: –remove-invalid-gene, –clean-mode strict, –threshold 0.98. A principal component analysis (PCA) was performed with the accessory genes (present in 5%–95%) in R Studio using the function *prcomp*. Gene content overrepresented in each clade was recovered using Scoary 2 (Roder et al., 2024), annotated using eggNOG-mapper v2 (Cantalapiedra et al., 2021) and gene context visualization of relevant regions was generated with Easyfig (Sullivan et al., 2011).

## 3 Results

### 3.1 Seventeen closed genomes of *G. parasuis* were assembled

The nanopore whole-genome sequencing of 17 *G. parasuis* isolates yielded a total of 364714 trimmed single ended reads, providing complete genome coverage between 48× and 359× per isolate, with a mean coverage of 155×. The coverage and read lengths were sufficient to assemble closed genome sequences for all isolates. The chromosome length ranged between 2,295,182 bp and 2,440,385 bp and the GC content between 39.55 and 40.05%. Two isolates (GPS_LSR012 and GPS_LSR013) contained three small circular plasmids of 9461 bp, 5673 bp and 3789 bp (Supplementary Table 2).

### 3.2 Seven different ST and five different serotypes were identified

**FIGURE 1:**
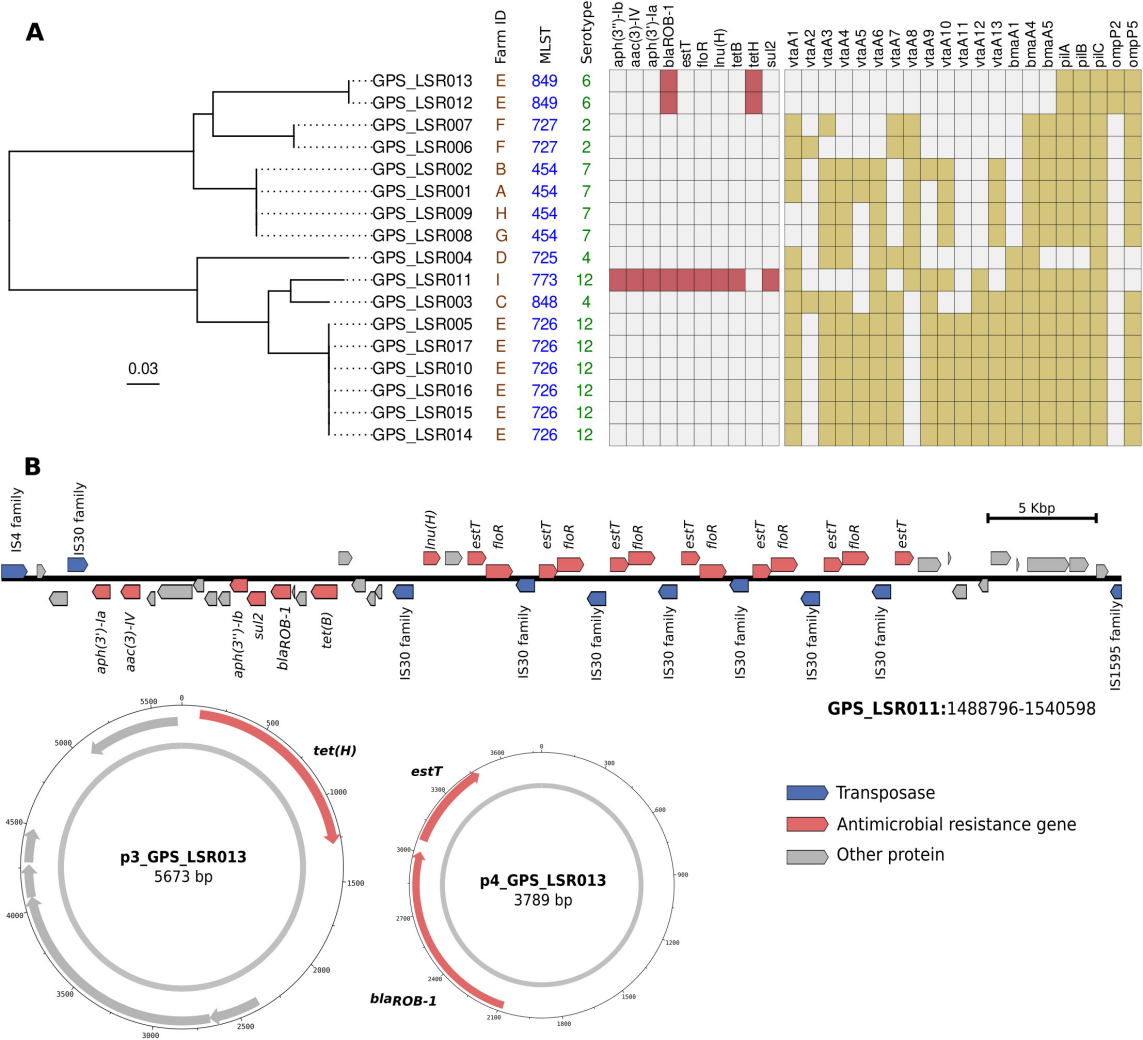
Genomic characteristics of Peruvian *G. parasuis* and its ARGs. **(A)** Maximum likelihood (ML) phylogenetic tree of 17 Peruvian isolates from this study with the MLST and serotype information. The presence of ARGs (red) and virulence factors (yellow) is shown in the matrix as colored squares. **(B)** Gene context of a genomic region of LSR011 isolate containing a cassette with nine different ARGs including tandem copies of *estT* and *florR* genes. Circular small plasmids containing ARGs are also depicted highlighting ARG genes in red.

Seven different STs were identified from genome sequence data. Most strains belonging to the same ST originated from the same farm, suggesting a degree of local clonal dissemination. Among the six novel STs identified (ST-725, ST-726, ST-727, ST-773, ST-848, and ST-849), ST-727 was characterized as a double-locus variant (DLV) of ST-548, while the remaining were classified as singletons (Supplementary Table 1). Each of these novel STs was restricted to a single farm (Figure 1A). Farm E contributed the largest number of isolates, comprising two different sequence types (ST726, *n* = 6; ST849, *n* = 2). Although ST-726 was most abundant in our dataset this is due to uneven sampling in Farm E. Instead, ST-454 was detected across four different farms suggesting that this clone is more widely disseminated and may have a greater capacity of farm-to-farm transmission.

We identified five different serotypes in our dataset. ST-726 (*n* = 6) and ST-773 (*n* = 1) belonged to serotype 12, while ST-725 (*n* = 1) and ST-848 (*n* = 1) belonged to serotype 4. The remaining serotypes were each associated with a single sequence type: serotype 7 with ST-454 (*n* = 4), serotype 2 with ST-727 (*n* = 2), and serotype 6 with ST-849 (*n* = 2).

### 3.3 Virulence-associated and ARGs

Most isolates (88.2%, 15/17) harbored at least one group 1 *vtaA* gene (Figure 1A). Regarding this group, the most frequently detected were *vtaA1* (76.5%, 13/17), *vtaA3* (76.5%, 13/17), *vtaA6* (76.5%, 13/17), *vtaA4* (70.6%, 12/17), and *vtaA7* (58.8%, 10/17) (Supplementary Table 1). The remaining group 1 *vtaA* genes were *vtaA9 (52.9%, 9/17), vtaA5 (*47.1%, 8/17)*, vtaA8* (47.1%, 8/17) and *vtaA2 (*11.8%, 2/17). Group 2 *vtaA* detected genes were *vtaA10* (64.7%, 11/17), and *vtaA11* (35.3%, 6/17) and within group 3 *vtaA* genes, *vtaA13* was detected in 64.7% (11/17) and *vtaA12* in 47.1% (8/17) of isolates. In addition, other putative virulence genes were screened. All isolates harbored the fimbrial *pilC* gene and most of the isolates (94.1%, 16/17) were positive for both the fimbrial *pilB* and *pilC* genes. Porin protein encoding *ompP2* and *ompP5* genes were present in 11.8% (2/17) and 88.2% (15/17) respectively. Regarding the monomeric autotransporter genes, *bmaA4* (88.2%, 15/17) and *bmaA5* (82.4%, 14/17) were the most frequent, while *bmaA1* (52.9%, 9/17) was the least frequent (Supplementary Table 1).

ARGs were absent in the majority of isolates (*n* = 14/17) (Figure 1A). Three isolates contained 10 different ARGs. The LRS011 (ST773) genome harbors a large genomic island (around 50 kb) carrying *bla_*ROB–*1_* (conferring resistance to betalactams), *aph(3′)-Ia, aac(3)-IVa* and *aph(3′)-Ib* (conferring resistance to different aminoglycosides), *sul2* (sulfonamide), *tetB* (tetracycline), *lnuH* (lincosamide), *estT* (macrolide) and *floR* (florfenicol-amphenicols). Interestingly, multiple copies of *estT* and *floR* were repeated in tandem interspaced by copies of IS30 transposase (Figure 1B). Additionally, *tetH*, *bla_*ROB–*1_* and *estT* were in two small plasmids of GPS_LSR012 and GPS_LSR013 (Figure 1B).

### 3.4 Phylogenetic analysis

**FIGURE 2:**
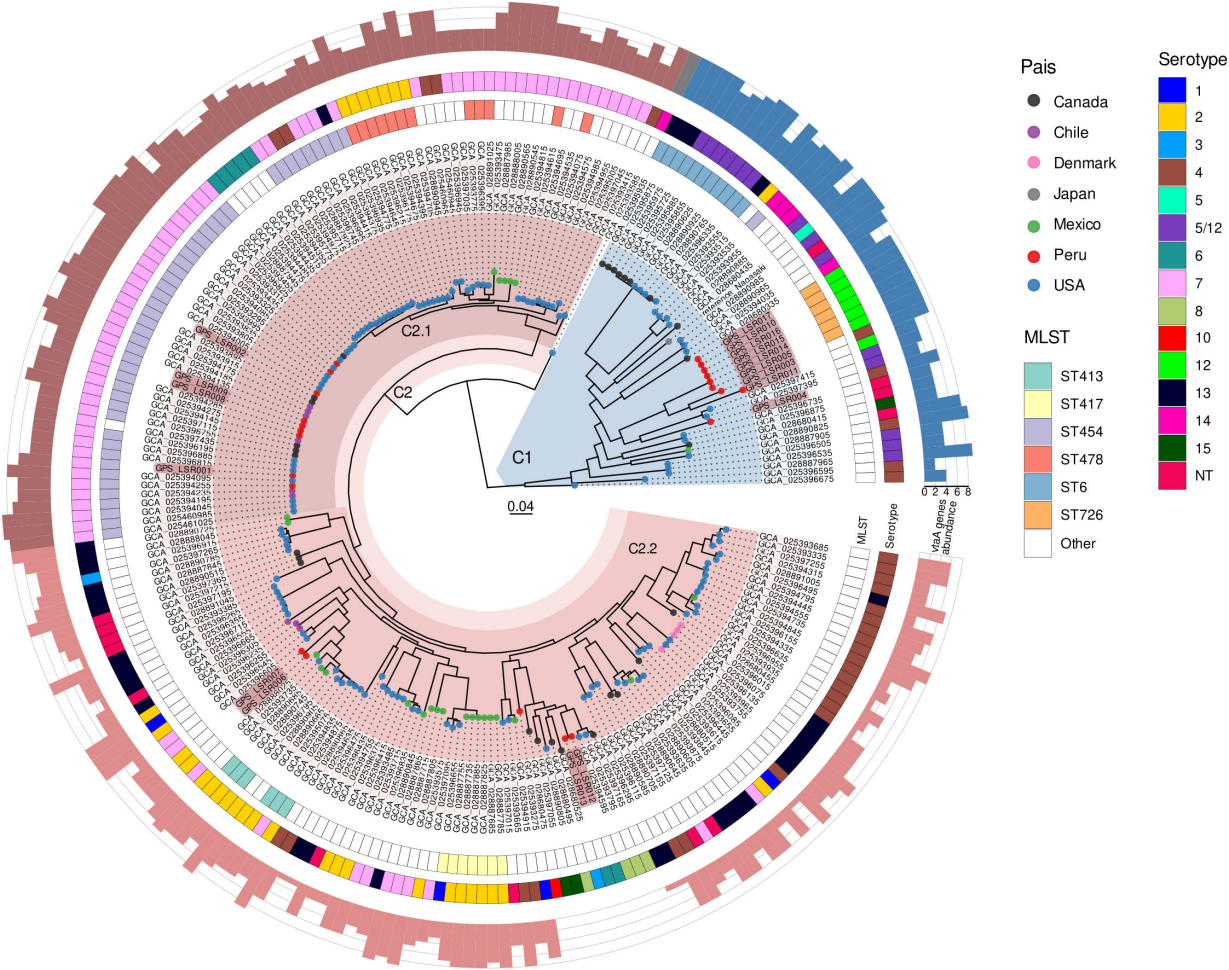
Phylogenetic tree of *G. parasuis*. Maximum likelihood phylogeny of core genome sequences for 234 *G. parasuis* isolates. Clades and subclades are highlighted. The label of isolates of this study are highlighted in pink. The color of the tippoint represents the country of origin. From the inner circle to the outer circle, the colors of the first circle represent the MLST, the second circle represents the serotype. The number of group 1 *vtaA* genes is represented by bar plot colored according to the clade.

The phylogenetic tree was constructed based on core-genome SNPs of 234 *G. parasuis* genomes including 17 novel genomes from Peru (Figure 2). The phylogenetic tree was resolved into two divergent clades (C1 and C2) in agreement with the classification proposed by Gong et al. (2024) based on comparative genomics of over 764 genomes. Clade C2 was further subdivided into two subclades, C2.1 and C2.2. The Peruvian isolates were distributed across both major clades, with nine isolates clustering within C1 (including six ST726-serotype12) and eight within C2, including four isolates (ST454-serotype7) assigned to subclade C2.1.

### 3.5 The pangenome of *G. parasuis* is open and clades show differential gene content

**FIGURE 3:**
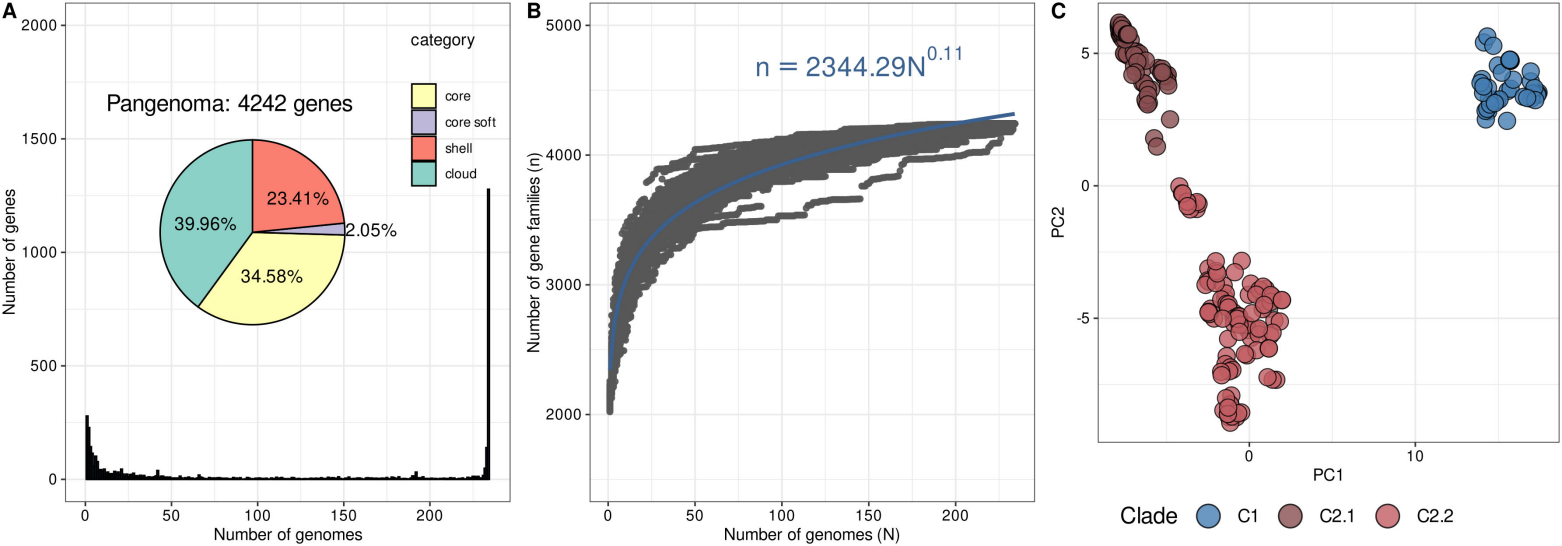
Pangenome statistics and PCA of accessory genome of *G. parasuis*. **(A)** The histogram shows the distribution of cloud, shell, and core genes and the pie chart displays the percentages. **(B)** Accumulation curve of the pagenome demonstrates the upward trend of the pan-gene families with additional genomes. Mathematical functions of pan-genome curves are also displayed in blue according to Heaps’ law. **(C)** PCA based on the accessory gene content matrix of 234 isolates, where each circle represents an isolate, colored according to its clade.

The pangenome of *G. parasuis* was predicted to comprise 4242 gene families including 1467 core genes (presented in >99% of isolates) which represent 34.6 % of the pangenome (Figure 3A). This core-genome size represents 69% of an average *G. parasuis* genome (2122 coding sequences). The accumulation curve of the pangenome and the power law regression suggest an open pangenome with alpha coefficient of 0.89 (Figure 3B). The PCA based on the presence/absence matrix of accessory genome (genes between 5 and 95% of isolates) shows a clear division between the accessory genes of C1 and C2 clades which suggest functional differences between the two clades (Figure 3C).

**FIGURE 4:**
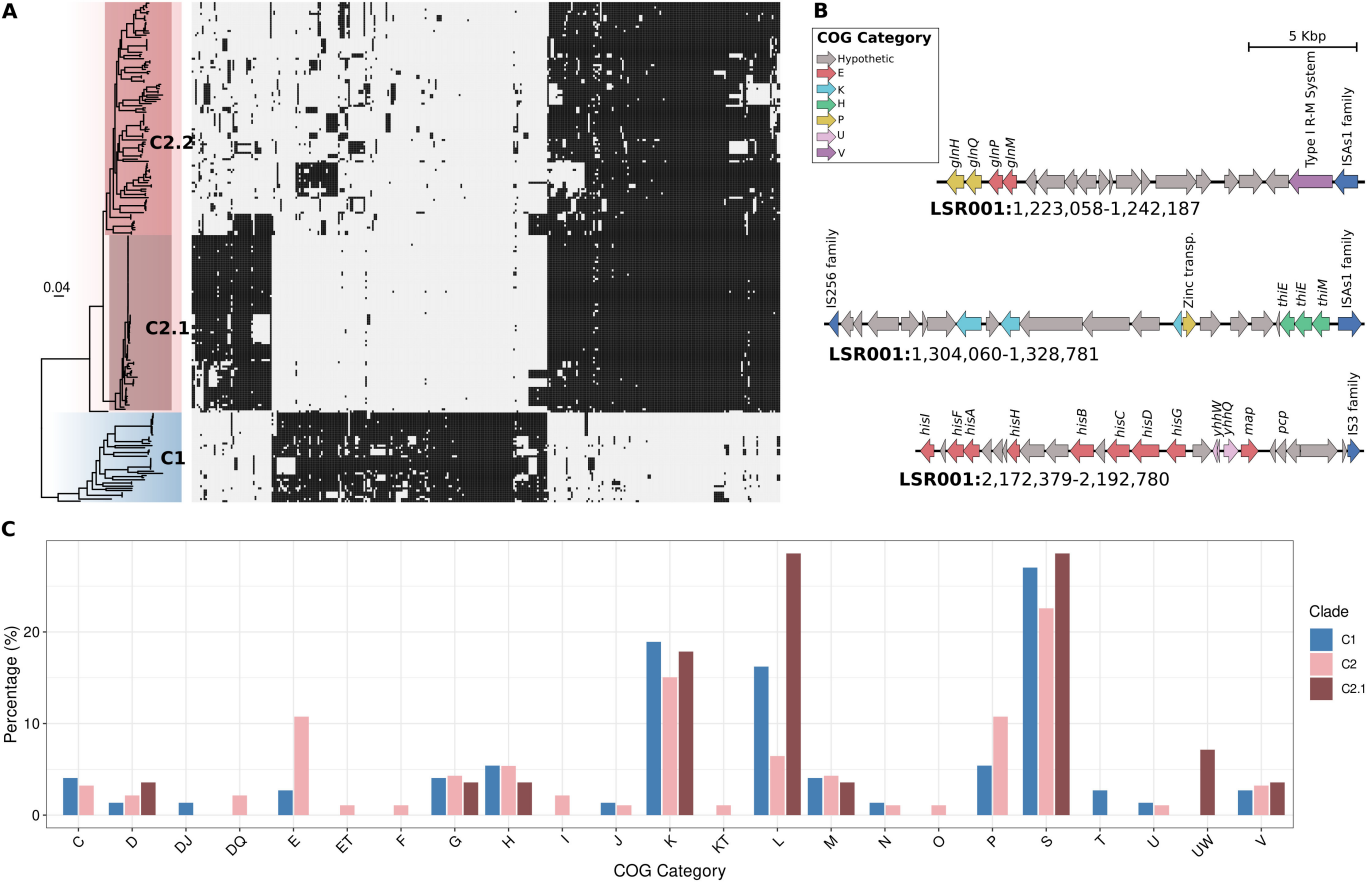
Identification of genes overrepresented in *G. parasuis* clades. **(A)** ML phylogenetic tree of 234 *G. parasuis* coupled to a presence absence matrix of differential gene content associated to clades C1, C2 and C2.1 identified by Scoary. **(B)** Schematic representation of three gene regions containing metabolic operons: *glnHMPQ, hisGDCBHAFI, thiDEM*, almost exclusive in C2 clade. **(C)** COG annotation of the overrepresented genes in C1, C2 and C2.1.

Scoary was used for the identification of genes either overrepresented or underrepresented in the clades C1, C2 and C2.1 (Figure 4A). This analysis identified 134 genes significantly overrepresented in the C1 clade (present in ≥80% of C1 isolates but in <20% of C2 isolates). Functional annotation using eggNOG-mapper assigned Clusters of Orthologous Groups (COG) categories to 74 of these genes, with categories S (Function unknown; 20 genes), K (Transcription; 14 genes), and L (Replication, recombination and repair; 12 genes) being the most prevalent (Figure 4C). In the C2 clade, Scoary identified 130 overrepresented genes, of which 99 were assigned to COG categories. Among these, categories S, K, P (Inorganic ion transport and metabolism), and E (Amino acid transport and metabolism) were more frequently represented. On the other hand, the subclade C2.1 contained 44 genes overrepresented, these were enriched mainly in S, K and L categories. Notably, in C2 we identified three genomic regions harboring operons associated with metabolic functions (Figure 4B): the *glnHMPQ* operon, involved in glutamine transport; the thiDEM operon, associated with thiamine biosynthesis; and the *hisGDCBHAFI* operon, related to histidine biosynthesis. These regions were flanked by insertion sequence (IS) elements, suggesting a horizontal transfer origin.

## 4 Discussion

Glässer’s disease remains one of the major diseases in pig farms worldwide (Aragon et al., 2019). Accurate diagnosis of pathogenic potential, antibiotic resistance levels and serotyping are necessary for adequate surveillance and control of Glässer’s disease (Costa-Hurtado et al., 2020). Therefore, whole genome sequencing represents a useful tool for the characterization of clinical *G. parasuis* isolates.

The *G. parasuis* sequenced in this study were highly diverse with seven different STs and five serotypes suggesting multiple clones circulating in the swine industry in Peru. Six novel STs detected supports the idea that *G. parasuis* populations are highly diverse, as previously reported (Wang et al., 2016; Turni et al., 2018). In 2018, Turni et al. (2018) identified 41 novel STs within 75 Australian isolates. Similarly, 66 novel STs were identified within 218 American isolates Mugabi et al. (2023), and 44 novel STs within 332 Chinese isolates Gong et al. (2024). On the other hand, the ST-454 which was first detected in the study by Mugabi et al. (2023), was detected in four different farms in Peru. This ST is associated with high mortality outbreaks, with signs of polyserositis and hyperacute disease. Additionally, five out of six *G. parasuis* from Peru sequenced by Mugabi et al. (2023) were of ST-454 further supporting its broad distribution across farms, years, and geographical regions (Gong et al., 2024). Interestingly, ST-726 was isolated from cases of polyserositis and pneumonia occurring on the same farm in different years, suggesting that ST-726 may represent an emerging ST in Peru. Nevertheless, a more extensive sampling is required to accurately determine the predominant STs circulating in the country.

We identified five different serotypes with serotype 7 (ST-454) and serotype 12 being more frequent. Serotyping has a significant epidemiological value, as well as for the development of bacterins targeting the most frequent serotypes in the region (Espíndola et al., 2019). Inactivated vaccines are most used for the control of Glässer disease. Two commercial vaccines are currently available in Peru: Porcilis Glässer (MSD Animal Health GmbH), based on serotype 5, and Hiprasuis Glässer (Hipra Laboratories, Spain), which contains a combination of serotypes 1 and 6. Although inactivated vaccines have demonstrated good homologous protection, their efficacy against heterologous strains has been inconsistent (Bak and Riising, 2002; Hau et al., 2025). Given that we identified different serotypes circulating on Peruvian farms, our data can inform the rational selection of strains for inclusion in autogenous vaccines, thereby maximizing protection.

Regarding virulence-associated genes, we focused on detecting group 1 *vtaA* genes, as evidence suggests they are good predictors of virulence (Pina et al., 2009; Olvera et al., 2010; Costa-Hurtado et al., 2012; Galofré-Milà et al., 2017). Almost all isolates in this study presented at least one group 1 *vtaA* gene, suggesting their pathogenic potential. Almost all isolates in this study presented at least one group 1 *vtaA* gene, suggesting their pathogenic potential. Although, at this time, no association has been demonstrated between the number of group 1 *vtaA* genes carried by an isolate and its virulence, we determined that isolates from clades C1 and C2.1 of the present study carried more group 1 *vtaA* genes and corresponded mostly with cases of systemic clinical disease. On the other hand, the absence of *vtaA* genes could be characteristic of non-pathogenic or commensal strains (Mugabi et al., 2023). However, in our study we found two isolates (GPS_LSR012 and GPS_LSR013) that lacked *vtaA* genes but were isolated from clinical cases, which coincides with Macedo et al. (2021) who found that some strains isolated from serotypes that are not usually associated with disease and lacking *vtaA* genes, such as serotype 6, were recovered from systemic disease. This could be explained by the fact that other environmental factors or co-infections could provide specific conditions for less virulent strains to cause disease (Turni et al., 2018; Van et al., 2019).

Other putative virulence genes were detected (fimbria, monomeric autotransporters and porin proteins). Most of these genes had frequencies above 82% (14/17) in the analyzed isolates. However, it has not yet been determined whether there is an association of these putative virulence genes with virulent or non-virulent isolates (Gong et al., 2024).

Although we did not detect ARGs in most of our isolates, one isolate contained a large cassette with nine different ARGs. Even though some isolates with high levels of ARGs have been reported previously (Sun et al., 2020, 2023; An et al., 2021), it is not as frequent as evidenced by Gong et al. (2024) who found that only 6.4% (49/764) of the isolates had more than six ARGs. The most interesting was that this cassette contained a tandem repetition of seven copies of *estT* and six copies of *floR*, conferring resistance to macrolides and florfenicol-amphenicols, respectively. The amplified region is flanked by two homologous IS30 family transposase copies, suggesting that the amplification may have been driven by these elements. Gene amplification can lead to high levels of antibiotic resistance as previously described in other bacteria species (Pavan et al., 2024) including amplification of *norA* gene in the CC398 lineage of *Staphylococcus aureus* resistant to ciprofloxacin (Papkou et al., 2020) and tandem amplification of chromosomal *mec* in *S. aureus* as driver of methicillin resistance in *S. aureus* (Gallagher et al., 2017).

We identified two isolates harboring *tetH, estT* and *bla*_*ROB–*1_ on two coexisting plasmids. Although the prevalence of these plasmids in our dataset was limited, the presence of small plasmids with similar structure in other *Pasteurellaceae* bacteria isolated from porcine revealed that this kind of plasmids are important cargos for the transmission resistance genes highlighting the need for continuous monitoring of these ARGs and mobile genetic elements in swine-associated pathogens (San Millan et al., 2009; Yao et al., 2023).

The diversity of *G. parasuis* was segregated into two main clades: C1 and C2 as also previously reported (Wan et al., 2020; Gong et al., 2024). We demonstrated that this segregation is also observed in terms of gene content (Figure 3C). We investigated differences in gene content between these clades identifying several genes overrepresented in each clade. Although most genes were identified into S, K and L COG categories, related with hypothetical functions and mobile genetic elements. Notably three metabolic operons (*glnHMPQ, hisGDCBHAFI, thiDEM*) were significantly enriched in the C2 lineage but almost absent in C1. While experimental validation is still needed, the absence of these operons suggests that while C2 strains retain the capacity to synthesize glutamine, histidine, and thiamine, C1 strains may be auxotrophic for these essential metabolites. Given that histidine and thiamine are critical for bacterial growth and survival (Fani et al., 1998), it raises the question of how C1 strains manage to persist. One possible explanation is that auxotrophic strains have adapted to specific host environments where these nutrients are readily available (D’Souza et al., 2014). This phenomenon has been observed in a closely related species *Haemophilus influenzae*, where histidine-auxotrophic strains exhibit enhanced survival in the human throat, an environment presumed to be rich in histidine. Similarly, methionine auxotrophy is commonly reported among *Pseudomonas aeruginosa* isolates from cystic fibrosis patients, suggesting adaptation to nutrient-rich host niches (Barth and Pitt, 1995). Due to limited metadata, we were unable to associate C1 and C2 strains with specific clinical manifestations or anatomical sites of isolation. Future studies should aim to assess whether these differences in metabolic capacity contribute to variations in pathogenicity, persistence or adaptation to different host niches.

## 5 Conclusion

This study suggests a considerable genetic diversity of *G. parasuis* circulating in the Peruvian swine industry reflected in the varied serotypes and novel STs identified within both C1 and C2 clades. The presence of diverse virulence factors and ARGs carried by mobile genetic elements highlights the need for continued genomic monitoring of the spread of *G. parasuis* in Perú. The results of this study contribute to increasing the knowledge of the molecular epidemiology and virulence of *G. parasuis* from pigs which is important to develop autogenous vaccine candidates and better control programs for Glässer’s disease. In addition, experimental evaluation could confirm the predicted metabolic differences between strains of C1 and C2 clades, providing insights into the pathogenesis and adaptation.

## Data Availability

The datasets presented in this study can be found in online repositories. The names of the repository/repositories and accession number(s) can be found in the article/Supplementary material.

## Footnotes

1
https://github.com/rrwick/Porechop

2
https://github.com/rrwick/Filtlong

3
https://github.com/rrwick/Autocycler

4
https://github.com/tseemann/abricate

5
https://github.com/tseemann/mlst

6
https://pubmlst.org/organisms/glaesserella-parasuis

## References

[B1] An J. Guo G. Yu D. Zhu K. Zhang C. Li Y. (2021). ICEHpsaHPS7, a novel multiple drug resistance integrative conjugative element in Glaesserella parasuis. *Antimicrob. Agents Chemother.* 65:e01716-20. 10.1128/AAC.01716-20 33199394 PMC7848986

[B2] Aragon V. Cerdà-Cuéllar M. Fraile L. Mombarg M. Nofrarías M. Olvera A. (2010). Correlation between clinico-pathological outcome and typing of Haemophilus parasuis field strains. *Vet. Microbiol.* 142 387–393. 10.1016/J.VETMIC.2009.10.025 19945233

[B3] Aragon V. Segalés J. Dan Tucker A. W. (2019). “Glásser’s disease”, in *Diseases of Swine*, 11th Edn, eds Zimmerman J. J. Locke A. K. Ramirez A. Schwartz K. J. Stevenson G. W. Zhang J. (Hoboken, NJ: Wiley), 844–853. 10.1002/9781119350927.ch54

[B4] Bak H. Riising H. J. (2002). Protection of vaccinated pigs against experimental infections with homologous and heterologous Hoemophilus parasuis. *Vet. Rec.* 151 502–505. 10.1136/VR.151.17.502 12430998

[B5] Barth A. L. Pitt T. L. (1995). Auxotrophic variants of *Pseudomonas aeruginosa* are selected from prototrophic wild-type strains in respiratory infections in patients with cystic fibrosis. *J. Clin. Microbiol.* 33 37–40. 10.1128/JCM.33.1.37-40.1995 7699062 PMC227875

[B6] Cantalapiedra C. P. Hernández-Plaza A. Letunic I. Bork P. Huerta-Cepas J. (2021). eggNOG-mapper v2: Functional annotation, orthology assignments, and domain prediction at the metagenomic scale. *Mol. Biol. Evol.* 38 5825–5829. 10.1093/MOLBEV/MSAB293 34597405 PMC8662613

[B7] Costa-Hurtado M. Ballester M. Galofré-Mil N. Darji A. Aragon V. (2012). VtaA8 and VtaA9 from Haemophilus parasuis delay phagocytosis by alveolar macrophages. *Vet. Res.* 43:57. 10.1186/1297-9716-43-57 22839779 PMC3462726

[B8] Costa-Hurtado M. Barba-Vidal E. Maldonado J. Aragon V. (2020). Update on Glässer’s disease: How to control the disease under restrictive use of antimicrobials. *Vet. Microbiol.* 242:108595. 10.1016/J.VETMIC.2020.108595 32122599

[B9] Costa-Hurtado M. Garcia-Rodriguez L. Lopez-Serrano S. Aragon V. (2019). Haemophilus parasuis VtaA2 is involved in adhesion to extracellular proteins. *Vet. Res.* 50 1–9. 10.1186/S13567-019-0687-2/FIGURES/5 31547880 PMC6755704

[B10] D’Souza G. Waschina S. Pande S. Bohl K. Kaleta C. Kost C. (2014). less is more: Selective advantages can explain the prevalent loss of biosynthetic genes in bacteria. *Evolution* 68 2559–2570. 10.1111/EVO.12468 24910088

[B11] Espíndola J. P. Balbinott N. Gressler L. T. Machado G. Klein C. S. Rebelatto R. (2019). Molecular serotyping of clinical strains of Haemophilus (Glaesserella) parasuis brings new insights regarding Glässer’s disease outbreaks in Brazil. *PeerJ* 7:e6817. 10.7717/PEERJ.6817 31198621 PMC6535215

[B12] Fani R. Mori E. Tamburini E. Lazcano A. (1998). Evolution of the structure and chromosomal distribution of histidine biosynthetic genes. *Origins Life Evol. Biosphere* 28 555–570. 10.1023/A:1006531526299/METRICS 9742729

[B13] Gallagher L. A. Coughlan S. Black N. S. Lalor P. Waters E. M. Wee B. (2017). Tandem amplification of the staphylococcal cassette chromosome mec element can drive high-level methicillin resistance in methicillin-resistant staphylococcus aureus. *Antimicrob. Agents Chemother.* 61:e00869-17. 10.1128/AAC.00869-17 28717043 PMC5571284

[B14] Galofré-Milà N. Correa-Fiz F. Lacouture S. Gottschalk M. Strutzberg-Minder K. Bensaid A. (2017). A robust PCR for the differentiation of potential virulent strains of Haemophilus parasuis. *BMC Vet. Res.* 13:124. 10.1186/S12917-017-1041-4 28482900 PMC5422950

[B15] Garrity G. Bell J. Lilburn T. (2005). *Bergey’s manual of systematic bacteriology*, Vol. 2B. New York: Springer, 10.1007/0-387-28022-7

[B16] Gong X. Cui Q. Zhang W. Shi Y. Zhang P. Zhang C. (2024). Genomic insight into the diversity of Glaesserella parasuis isolates from 19 countries. *mSphere*. 9:e00231-24. 10.1128/MSPHERE.00231-24 39194201 PMC11423579

[B17] Gurevich A. Saveliev V. Vyahhi N. Tesler G. (2013). QUAST: Quality assessment tool for genome assemblies. *Bioinformatics* 29 1072–1075. 10.1093/BIOINFORMATICS/BTT086 23422339 PMC3624806

[B18] Hau S. J. Luan S. L. Weinert L. A. Langford P. R. Rycroft A. Wren B. W. (2025). Capsular immunity is necessary for protection against some but not all strains of Glaesserella parasuis. *Vet. Microbiol.* 305:110509. 10.1016/J.VETMIC.2025.110509 40250105 PMC12094177

[B19] Howell K. J. Peters S. E. Wang J. Hernandez-Garcia J. Weinert L. A. Luan S. L. (2015). Development of a multiplex PCR assay for rapid molecular serotyping of Haemophilus parasuis. *J. Clin. Microbiol.* 53:3812. 10.1128/JCM.01991-15 26424843 PMC4652097

[B20] Hu J. Wang Z. Sun Z. Hu B. Ayoola A. O. Liang F. (2024). NextDenovo: An efficient error correction and accurate assembly tool for noisy long reads. *Genome Biol.* 25 1–19. 10.1186/S13059-024-03252-4/FIGURES/3 38671502 PMC11046930

[B21] Jain C. Rodriguez-R L. M. Phillippy A. M. Konstantinidis K. T. Aluru S. (2018). High throughput ANI analysis of 90K prokaryotic genomes reveals clear species boundaries. *Nat. Commun.* 9:5114. 10.1038/s41467-018-07641-9 30504855 PMC6269478

[B22] Jia A. Zhou R. Fan H. Yang K. Zhang J. Xu Y. (2017). Development of serotype-specific PCR assays for typing of haemophilus parasuis isolates circulating in Southern China. *J. Clin. Microbiol.* 55:3249. 10.1128/JCM.00688-17 28878007 PMC5654909

[B23] Kielstein P. Rapp-Gabrielson V. J. (1992). Designation of 15 serovars of Haemophilus parasuis on the basis of immunodiffusion using heat-stable antigen extracts. *J. Clin. Microbiol.* 30 862–865. 10.1128/JCM.30.4.862-865.1992 1572971 PMC265175

[B24] Kille B. Nute M. G. Huang V. Kim E. Phillippy A. M. Treangen T. J. (2024). Parsnp 2.0: Scalable core-genome alignment for massive microbial datasets. *Bioinformatics* 40:btae311. 10.1093/BIOINFORMATICS/BTAE311 38724243 PMC11128092

[B25] Kolmogorov M. Yuan J. Lin Y. Pevzner P. A. (2019). Assembly of long, error-prone reads using repeat graphs. *Nat. Biotechnol.* 37 540–546. 10.1038/s41587-019-0072-8 30936562

[B26] Koren S. Walenz B. P. Berlin K. Miller J. R. Bergman N. H. Phillippy A. M. (2017). Canu: Scalable and accurate long-read assembly via adaptive k-mer weighting and repeat separation. *Genome Res.* 27 722–736. 10.1101/GR.215087.116 28298431 PMC5411767

[B27] Li H. (2016). Minimap and miniasm: Fast mapping and de novo assembly for noisy long sequences. *Bioinformatics* 32 2103–2110. 10.1093/BIOINFORMATICS/BTW152 27153593 PMC4937194

[B28] Macedo N. Gottschalk M. Strutzberg-Minder K. Van C. N. Zhang L. Zou G. (2021). Molecular characterization of Glaesserella parasuis strains isolated from North America, Europe and Asia by serotyping PCR and LS-PCR. *Vet. Res.* 52 1–10. 10.1186/S13567-021-00935-9/TABLES/8 33980312 PMC8117636

[B29] Macedo N. Rovira A. Torremorell M (2015). Haemophilus parasuis: Infection, immunity and enrofloxacin. *Vet. Res.* 46 1–6. 10.1186/S13567-015-0263-3/METRICS 26511717 PMC4625873

[B30] Markey B. Leonard F. Archambault M. Cullinane A. Maguire D. (2013). *Clinical veterinary microbiology*, 2nd Edn. Amsterdam: Elsevier.

[B31] Martínez-Moliner V. Soler-Llorens P. Moleres J. Garmendia J. Aragon V. (2012). Distribution of genes involved in sialic acid utilization in strains of Haemophilus parasuis. *Microbiology* 158 2117–2124. 10.1099/MIC.0.056994-0 22609756

[B32] Minh B. Q. Schmidt H. A. Chernomor O. Schrempf D. Woodhams M. D. Von Haeseler A. (2020). IQ-TREE 2: New models and efficient methods for phylogenetic inference in the genomic era. *Mol. Biol. Evol.* 37 1530–1534. 10.1093/MOLBEV/MSAA015 32011700 PMC7182206

[B33] Mugabi R. Silva A. P. S. P. Hu X. Gottschalk M. Aragon V. Macedo N. R. (2023). Molecular characterization of Glaesserella parasuis strains circulating in North American swine production systems. *BMC Vet. Res.* 19:135. 10.1186/S12917-023-03698-X/FIGURES/4 37641044 PMC10464461

[B34] Mullins M. A. Register K. B. Brunelle B. W. Aragon V. Galofré-Mila N. Bayles D. O. (2013). A curated public database for multilocus sequence typing (MLST) and analysis of Haemophilus parasuis based on an optimized typing scheme. *Vet. Microbiol.* 162 899–906. 10.1016/J.VETMIC.2012.11.019 23218953

[B35] Oliveira S. Pijoan C. (2004). Haemophilus parasuis: New trends on diagnosis, epidemiology and control. *Vet. Microbiol.* 99 1–12. 10.1016/j.vetmic.2003.12.001 15019107

[B36] Olvera A. Pina S. Pérez-Simó M. Oliveira S. Bensaid A. (2010). Virulence-associated trimeric autotransporters of Haemophilus parasuis are antigenic proteins expressed in vivo. *Vet. Res.* 41:26. 10.1051/VETRES/2009074 19995512 PMC2820231

[B37] Papkou A. Hedge J. Kapel N. Young B. MacLean R. C. (2020). Efflux pump activity potentiates the evolution of antibiotic resistance across S. aureus isolates. *Nat. Commun.* 11:3970. 10.1038/s41467-020-17735-y 32769975 PMC7414891

[B38] Pavan K. Silva T. Khare A. (2024). Antibiotic resistance mediated by gene amplifications. *npj Antimicrob Resist.* 2:35. 10.1038/s44259-024-00052-5 39843582 PMC11721125

[B39] Pina S. Olvera A. Barceló A. Bensaid A. (2009). Trimeric autotransporters of haemophilus parasuis: Generation of an extensive passenger domain repertoire specific for pathogenic strains. *J. Bacteriol.* 191 576–587. 10.1128/JB.00703-08/SUPPL_FILE/TABLE_S2.ZIP 19011035 PMC2620822

[B40] Roder T. Pimentel G. Fuchsmann P. Stern M. T. von, Ah U. (2024). Scoary2: Rapid association of phenotypic multi-omics data with microbial pan-genomes. *Genome Biol.* 25:93. 10.1186/S13059-024-03233-7/TABLES/1 38605417 PMC11007987

[B41] Sack M. Baltes N. (2009). Identification of novel potential virulence-associated factors in Haemophilus parasuis. *Vet. Microbiol.* 136 382–386. 10.1016/J.VETMIC.2008.11.008 19117700

[B42] San Millan A. Escudero J. A. Gutierrez B. Hidalgo L. Garcia N. Llagostera M. (2009). Multiresistance in Pasteurella multocida is mediated by coexistence of small plasmids. *Antimicrob. Agents Chemother.* 53 3399–3404. 10.1128/AAC.01522-08/ASSET/D68F657D-4A08-4ACC-8166-43199BFEA181/ASSETS/GRAPHIC/ZAC0080983520003.JPEG 19528282 PMC2715648

[B43] Seemann T. (2014). Prokka: Rapid prokaryotic genome annotation. *Bioinformatics* 30 2068–2069. 10.1093/BIOINFORMATICS/BTU153 24642063

[B44] Sullivan M. J. Petty N. K. Beatson S. A. (2011). Easyfig: A genome comparison visualizer. *Bioinformatics* 27 1009–1010. 10.1093/BIOINFORMATICS/BTR039 21278367 PMC3065679

[B45] Sun H. R. Cui X. D. Liu X. K. Li S. H. Yi K. F. Pan Y. S. (2020). Molecular characterization of a novel integrative conjugative element ICE Hpa1 in Haemophilus parasuis. *Front. Microbiol.* 11:1884. 10.3389/FMICB.2020.01884 32903523 PMC7438473

[B46] Sun H. Yang Y. Yi K. Zhang M. Luo X. He D. (2023). ICEGpa1804, a novel integrative and conjugative element carrying eight resistance genes, identified in Glaesserella parasuis. *Int. J. Antimicrob. Agents* 61:106740. 10.1016/J.IJANTIMICAG.2023.106740 36736498

[B47] Tadjine M. Mittal K. R. Bourdon S. Gottschalk M. (2004). Development of a new serological test for serotyping Haemophilus parasuis isolates and determination of their prevalence in North America. *J. Clin. Microbiol.* 42 839–840. 10.1128/JCM.42.2.839-840.2004 14766867 PMC344452

[B48] Tonkin-Hill G. MacAlasdair N. Ruis C. Weimann A. Horesh G. Lees J. A. (2020). Producing polished prokaryotic pangenomes with the Panaroo pipeline. *Genome Biol.* 21 1–21. 10.1186/S13059-020-02090-4/FIGURES/7 PMC7376924 32698896

[B49] Turni C. Pyke M. Blackall P. J. (2010). Validation of a real-time PCR for Haemophilus parasuis. *J. Appl. Microbiol.* 108 1323–1331. 10.1111/J.1365-2672.2009.04526.X 19778350

[B50] Turni C. Singh R. Blackall P. J. (2018). Virulence-associated gene profiling, DNA fingerprinting and multilocus sequence typing of Haemophilus parasuis isolates in Australia. *Aust. Vet. J.* 96 196–202. 10.1111/AVJ.12705 29878333

[B51] Van C. N. Thanh T. V. T. Zou G. Jia M. Wang Q. Zhang L. (2019). Characterization of serotypes and virulence genes of Haemophilus parasuis isolates from Central Vietnam. *Vet. Microbiol.* 230 117–122. 10.1016/J.VETMIC.2019.02.008 30827376

[B52] Wan X. Li X. Osmundson T. Li C. Yan H. (2020). Whole-genome sequence analyses of Glaesserella parasuis isolates reveals extensive genomic variation and diverse antibiotic resistance determinants. *PeerJ* 2020:e9293. 10.7717/PEERJ.9293/SUPP-64 32607281 PMC7316082

[B53] Wang L. Ma L. Liu Y. Gao P. Li Y. Li X. (2016). Multilocus sequence typing and virulence analysis of Haemophilus parasuis strains isolated in five provinces of China. *Infect. Genet. Evol.* 44 228–233. 10.1016/J.MEEGID.2016.07.015 27431332

[B54] Wick R. R. Howden B. P. Stinear T. P. (2025). Autocycler: Long-read consensus assembly for bacterial genomes. *bioRxiv [Preprint]* 10.1101/2025.05.12.653612 PMC12460055 40875535

[B55] Yao X. Song Q. Zhu W. Wei J. Shao D. Liu K. (2023). Characterization of small plasmids carrying florfenicol resistance gene floR in Actinobacillus pleuropneumoniae and Pasteurella multocida isolates from swine in China. *Front. Vet. Sci.* 10:1084491. 10.3389/FVETS.2023.1084491/BIBTEX 36793377 PMC9922843

[B56] Yu G. (2020). Using ggtree to visualize data on tree-like structures. *Curr. Protoc. Bioinformatics* 69:e96. 10.1002/CPBI.96 32162851

[B57] Zhang B. Tang C. Liao M. Yue H. (2014). Update on the pathogenesis of Haemophilus parasuis infection and virulence factors. *Vet. Microbiol.* 168 1–7. 10.1016/J.VETMIC.2013.07.027 23972951

